# Nationwide cohort study on the risk of high‐grade cervical dysplasia and carcinoma after conservative treatment or hysterectomy for adenocarcinoma in situ

**DOI:** 10.1002/ijc.35237

**Published:** 2024-11-04

**Authors:** Mirte Schaafsma, Teska N. Schuurman, Pien Kootstra, Deli Issa, Ivo Hermans, Maaike C. G. Bleeker, Petra L. M. Zusterzeel, Ruud L. M. Bekkers, Albert G. Siebers, Constantijne H. Mom, Nienke E. van Trommel

**Affiliations:** ^1^ Department of Gynecologic Oncology, Center of Gynecologic Oncology Amsterdam Antoni van Leeuwenhoek/Netherlands Cancer Institute Amsterdam The Netherlands; ^2^ Department of Pathology, Amsterdam UMC Vrije Universiteit Amsterdam Amsterdam The Netherlands; ^3^ Biomarkers and Imaging Cancer Center Amsterdam Amsterdam The Netherlands; ^4^ Department of Gynecologic Oncology, Center of Gynecologic Oncology Amsterdam, Amsterdam UMC Vrije Universiteit Amsterdam Amsterdam The Netherlands; ^5^ Department of Gynecology Catharina Hospital Eindhoven The Netherlands; ^6^ Department of Obstetrics and Gynecology Radboud University Medical Center Nijmegen The Netherlands; ^7^ Department of Obstetrics and Gynecology, Maastricht University Medical Center and GROW‐School for Oncology and Reproduction Maastricht University Maastricht The Netherlands; ^8^ Palga, the Dutch nationwide pathology databank Houten The Netherlands

**Keywords:** adenocarcinoma in situ, cervical cancer, surgical treatment

## Abstract

Internationally, little consensus exists about the best treatment for cervical adenocarcinoma in situ (AIS). This study aimed to determine the incidence of recurrent high‐grade cervical dysplasia and development of local cervical cancer after treatment for AIS. This nationwide, retrospective cohort study included patients with AIS, who were treated by a large loop excision of the transformation zone (LLETZ), cold‐knife conization (CKC), or hysterectomy between January 1, 1990 and December 31, 2021 in the Netherlands. Pathology reports were retrieved from the Dutch Nationwide Pathology Databank (Palga). Primary outcomes were the cumulative incidences of high‐grade cervical dysplasia (cervical intraepithelial neoplasia grade 2 or 3, and AIS) and local cervical cancer up to 20 years after primary treatment. In total, 4243 patients with AIS were included. The primary treatment was a LLETZ, CKC, or hysterectomy in 1593, 2118, and 532 patients, respectively. The incidence of recurrent high‐grade cervical dysplasia after LLETZ (10.5%; 95%CI: 8.6–12.3) was higher than after CKC (5.5%; 95%CI: 4.4–6.6, *p* <.0001). When a radical excision, that is, surgical margins free of dysplasia at end of treatment, was achieved, the incidence of recurrent high‐grade dysplasia and local cervical cancer did not differ between LLETZ (5.6% [95%CI: 3.3–7.9] and 1.9% [95%CI: 0–4.4]) and CKC (4.7% [95%CI: 3.5–5.8], *p* = .631 and 1.5% [95%CI: 0.7–2.3], *p* = .918). After hysterectomy, none of the patients developed cervical dysplasia or local cervical cancer. Conservative treatment for AIS can be considered a safe and final treatment modality when a radical excision is achieved.

## Graphical abstract

What's new?

There are various strategies available for the treatment of cervical adenocarcinoma in situ (AIS). Which approach is the most effective for preventing progression to cervical carcinoma, however, remains uncertain. In this study, the authors compared outcomes of AIS patients treated with either large loop excision of the transformation zone (LLETZ), cold‐knife conization (CKC), or hysterectomy. Following radical LLETZ or radical CKC, cervical high‐grade dysplasia and local cervical cancer incidence were similar, while no malignancies developed after hysterectomy. The data indicate that conservative treatment with successful radical excision can be an effective long‐term treatment modality for AIS.

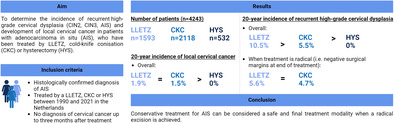

## 1 INTRODUCTION

Cervical adenocarcinoma in situ (AIS) is a non‐invasive precursor lesion of cervical adenocarcinoma. Treatment options are either a hysterectomy or conservative by a large loop excision of the transformation zone (LLETZ) or cold‐knife conization (CKC). In current literature, there is little consensus about the best treatment strategy for AIS, which leads to conflicting recommendations in international guidelines.^1^, ^2^, ^3^, ^4^, ^5^ In some American and European guidelines, a simple hysterectomy is the treatment of choice, either as primary treatment, or complementary, after initial conservative surgery and completion of childbearing.^1^, ^2^, ^6^ The main rationale for this advice is the association of AIS with multifocal lesions, reported in 7%–53% of cases,^7^, ^8^, ^9^, ^10^, ^11^ and the chance of residual AIS, or even occult adenocarcinoma, despite negative surgical margins.^1^, ^8^ The risk of residual AIS, even with negative margins on the excisional specimen, is estimated at 20%.^1^, ^12^

In the Netherlands, the standard treatment for cervical AIS is either a LLETZ, CKC, or hysterectomy, with a preference for CKC over LLETZ. The advice for a complementary hysterectomy after the completion of childbearing is not included in the national guideline, since low recurrence rates after conservative surgery are reported.^3^

Since many patients with AIS are of childbearing age, conservative treatment is often indicated. However, there is controversy about the best conservative management of AIS in terms of oncological outcome. Small retrospective studies comparing both surgical procedures show conflicting results regarding the risk of positive margins, residual lesions, or recurrences, with either similar effectiveness^13^, ^14^, ^15^ or favoring CKC over LLETZ.^7^, ^11^, ^13^, ^14^, ^15^, ^16^ Additionally, the possible negative impact on obstetric outcomes after both LLETZ and CKC should be taken into account when choosing the optimal treatment.^17^ The risk of preterm delivery is associated with the depth and amount of excised volume of the cervix, starting at 0.5cc or more, and is reported to be higher after CKC than after LLETZ.^17^, ^18^, ^19^ The low level of evidence currently available, and the subsequent lack of consensus about the optimal treatment strategy for patients with AIS, indicates the need for a large population‐based study with long follow‐up.

This study aimed to determine the cumulative incidence of recurrent high‐grade cervical dysplasia and development of local cervical cancer in patients with AIS, who have been treated by LLETZ, CKC, or hysterectomy. Additionally, the association between treatment modality and oncologic outcome was analyzed.

## 2 METHODS

A nationwide, retrospective cohort study was performed using data from pathology reports obtained from the Dutch Nationwide Pathology Databank (Palga) and mortality data from the Central Bureau of Genealogy (CBG).^20^ The STrengthening the Reporting of OBservational studies in Epidemiology (STROBE) guidelines were followed for reporting.^21^

### 2.1 Patients

Patients diagnosed with severe cervical atypical glandular neoplasia or cervical AIS between January 1, 1990 and December 31, 2021 were identified by Palga. Pathology reports were reviewed to determine eligibility. Inclusion criteria were a histologically confirmed first diagnosis of AIS, and primary treatment by LLETZ, CKC, or total hysterectomy (including the cervix). Exclusion criteria were a history of cervical, ovarian, or endometrial cancer, or the diagnosis of cervical cancer within 3 months after primary treatment of AIS. The cut‐off of 3 months was chosen, because we assumed that occult cervical cancer detected within 3 months after primary treatment of AIS was already present at time of treatment. The other exclusion criteria were patients with missing pathology reports or with an interval of more than 1 year between AIS diagnosis and treatment.

### 2.2 Outcomes

The primary outcome was the cumulative incidence of recurrent high‐grade cervical dysplasia (cervical intraepithelial neoplasia [CIN] grade 2 or 3, and AIS), and the cumulative incidence of local cervical cancer up to 20 years after primary treatment for AIS. Primary treatment was defined as the most invasive surgical procedure, that is, a hysterectomy was considered most invasive, followed by CKC and LLETZ. The end of primary treatment was set at the date on which the last surgical procedure to treat AIS or residual high‐grade cervical dysplasia was performed. Residual high‐grade dysplasia was defined as CIN2‐3 or AIS in a subsequent excision within 3 months after first cervical excision for AIS. Recurrent high‐grade cervical dysplasia, hereafter called high‐grade dysplasia, was defined as a histological diagnosis of CIN2‐3 or AIS after the end of primary treatment. Development of local cervical cancer was defined as a histological diagnosis of squamous cell carcinoma, adeno(squamous)carcinoma, or carcinoma not otherwise specified (NOS) located at the cervix or vaginal vault. We primarily analyzed these composite outcome measures, since all (pre)malignant outcomes are relevant and possible indications for retreatment, affecting future obstetric outcomes in an equal matter. Additionally, subgroup analyses were performed, using the outcomes recurrent AIS, CIN2, and CIN3, cervical cancer of any histotype at any location (local, regional, or distant), adeno(squamous)carcinoma at any location, and squamous cell carcinoma at any location.

The secondary outcome was the association between treatment modality and primary outcome, adjusted for potential confounders. To identify potential confounders, a literature search^10^, ^13^, ^22^, ^23^, ^24^, ^25^, ^26^, ^27^ was performed and a directed acyclic graph (DAG^28^) was construed in a consensus meeting with experts (Supplemental Figure S1). The potential mediator of the association was surgical radicality. Potential confounders were age and coexisting CIN lesions.

Surgical radicality was based on the margin status of the last surgical excision of the primary treatment. It was subdivided into three categories: radical excision, when dysplasia (AIS, CIN1‐3, and dysplasia NOS) was absent in the surgical margins, irradical excision when dysplasia was present in surgical margins, and unclear when surgical margins were not reported or reported as doubtful or unclear. When no dysplasia was identified in the specimen of the last surgical excision, the treatment was assumed to be radical.

Coexisting CIN lesions were defined as CIN1‐3 or dysplasia NOS within 3 months before AIS diagnosis or between AIS diagnosis and end of primary treatment.

### 2.3 Statistical analysis

Descriptive statistics were summarized by medians and interquartile ranges (IQR) for numerical values and by proportions and percentages for categorical variables. For numerical values normality was assessed by visual inspection and Schoenfeld's residuals. Comparisons between treatment groups were done by the ANOVA test for normally distributed numerical variables, the Kruskal–Wallis test for non‐normally distributed numerical variables and the chi‐squared test for categorical variables. Comparisons between numerical variables of two groups were done by the independent *t*‐test or the Mann–Whitney U test depending on normality. General characteristics stratified by year of AIS treatment were compared between four time cohorts using the trend chi‐squared test. The association between the number of AIS diagnoses and the year of diagnosis was evaluated by linear regression analysis.

Intention‐to‐treat analyses were done by Kaplan–Meier analyses. Time‐to‐event was defined as the time between the end of primary treatment and the event. The end of follow‐up was set at the date of completion of data collection (July 10, 2023), unless patients were registered as deceased in the CBG, in which case date of death was used. Additionally, event‐specific censoring was done: for high‐grade dysplasia, patients were censored when they were diagnosed with cervical cancer; for development of adeno(squamous)carcinoma or squamous cell carcinoma, patients were censored when they were diagnosed with cervical cancer of another histotype; for development of local cervical cancer, patients were censored when they were diagnosed with regional or distant metastases. All incidences were stratified by primary treatment, and separately for surgical radicality and year of treatment.

Univariable and multivariable cox regression analyses were performed to assess the association between treatment and primary outcome up to 20 years follow‐up. The association between treatment and high‐grade dysplasia was adjusted in the first multivariable model for surgical radicality and effect modification between time and radicality and in the second multivariable model for radicality, effect modification between time and radicality, age, and coexisting CIN lesions. The mediation effect of radicality was calculated by comparing the beta‐coefficients of treatment between the univariable model and the first multivariable model. The association between treatment and development of local cervical cancer was assessed using univariable and multivariable cox regression analysis. In the latter, the association was adjusted for radicality. The assumption of proportional hazards was fulfilled for all models.

All statistical analyses were performed in R (R, Vienna, Austria), version 4.2.0, specifically using the packages survival and stats.

## 3 RESULTS

**FIGURE 1:**
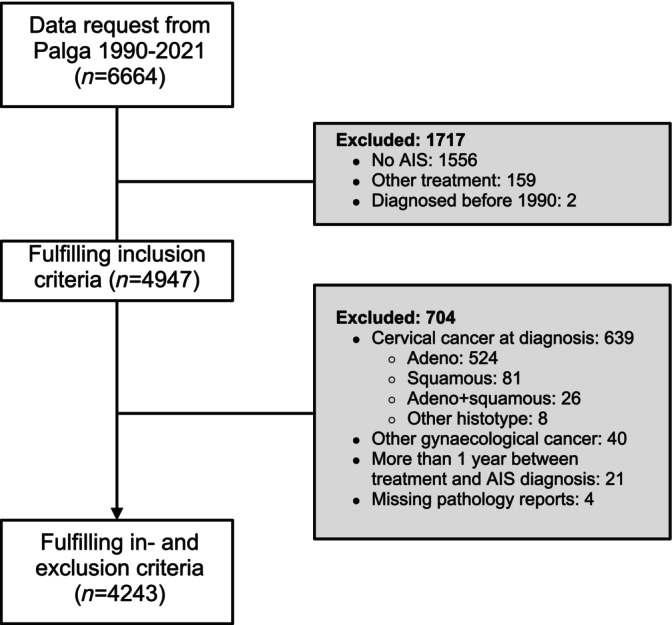
Flow diagram of included patients.

In total, 6664 patients were screened for eligibility after which 4243 patients (63.7%) were included in this study. Of these patients, 42,767 pathology reports were analyzed (Figure 1). Of the included 4243 patients, 1593 patients (37.5%) received a LLETZ, 2118 patients (49.9%) a CKC, and 532 patients (12.5%) a hysterectomy as primary treatment (Table 1). Supplemental Figure S2 shows the number and type of surgical excisions performed within each primary treatment group. In the LLETZ group, 159 of 1593 patients (10%) received more than one LLETZ. In the CKC group, the CKC was preceded by a LLETZ in 541 of 2118 patients (25.5%). The majority of the patients in the hysterectomy group (386 of 532 patients, 72.6%), received a LLETZ or CKC prior to the hysterectomy. Treatment by LLETZ compared to CKC was more often irradical (317 of 1593 patients, 19.9% vs. 211 of 2118 patients, 10%; *p* <.0001), or of unclear radicality (621 of 1593 patients, 39% vs. 247 of 2118 patients, 11.7%; *p* <.0001).

**TABLE 1:** Patients' characteristics.

		LLETZ (*n* = 1593)	CKC (*n* = 2118)	Hysterectomy (*n* = 532)	*p‐value*
Age (median [IQR])		34 [30–40]	35 [30–40]	43 [39–50]	<.001^a^
Year of treatment (%)	1990–1994	23 (1.4)	190 (9.0)	58 (10.9)	<.001
	1995–1999	88 (5.5)	252 (11.9)	55 (10.3)	
	2000–2004	132 (8.3)	230 (10.9)	49 (9.2)	
	2005–2009	185 (11.6)	265 (12.5)	62 (11.7)	
	2010–2014	320 (20.1)	377 (17.8)	79 (14.8)	
	2015–2019	684 (42.9)	643 (30.4)	178 (33.5)	
	≥2020	161 (10.1)	161 (7.6)	51 (9.6)	
Number of surgical procedures (%)	1	1434 (90.0)	1508 (71.2)	146 (27.4)	<.001
	2	153 (9.6)	601 (28.4)	344 (64.7)	
	3–4	6 (0.4)	9 (0.4)	42 (7.9)	
Coexisting CIN (%)	CIN1/CIN NOS	136 (8.5)	187 (8.8)	37 (7.0)	<.001
	CIN2	201 (12.6)	250 (11.8)	70 (13.2)	
	CIN3	793 (49.8)	954 (45.0)	197 (37.0)	
	None	463 (29.1)	727 (34.3)	228 (42.9)	
Radicality (%)	Radical	655 (41.1)	1660 (78.4)	509 (95.7)	<.001
	Irradical	317 (19.9)	211 (10.0)	1 (0.2)	
	Unclear	621 (39.0)	247 (11.7)	22 (4.1)	
Follow‐up—years (median [IQR])		7.7 [4.8–13.9]	11.5 [5.9–21.2]	10.1 [5.3–19.4]	<.001
Time between diagnosis of AIS and primary treatment—days (median [IQR])		0 [0–28]	29 [14–48]	61 [31–100]	<.001

Abbreviations: AIS, adenocarcinoma in situ; CIN, cervical intraepithelial neoplasia; CKC, cold‐knife conization; IQR, interquartile range; LLETZ, large loop excision of the transformation zone; NOS, not otherwise specified.

^a^ Based on log‐transformed data tested by the ANOVA test.

Overall, the median follow‐up was 9.8 years (IQR 5.3–18.2). Follow‐up was longer after CKC (median 11.5 years, IQR 5.9–21.2) compared to LLETZ (median 7.7 years, IQR 4.8–13.9; *p* = <.0001) and hysterectomy (median 10.1 years, IQR 5.3–19.4; *p* = .022). During follow‐up after conservative treatment, 3403 of 3711 patients (91.7%) received follow‐up by cervical cytology. The average number of cytology tests per patient was 6 (median 5, IQR 3–7). High‐risk human papillomavirus (HPV) testing in the follow‐up after LLETZ and CKC were available for 2767 of 3711 patients (74.6%), with an average of 3 tests per patient (median 2, IQR 1–4). Following primary treatment by LLETZ or CKC, a hysterectomy during follow‐up was performed in 62 of 1593 (3.9%) patients and in 140 of 2118 (6.6%) patients, respectively.

Supplemental Table S1 describes the general characteristics of the included patients stratified by year of AIS treatment. Between 1990 and 2020, the number of AIS diagnoses increased on average with a multiplication factor of 1.06 each year (95% CI: 1.06–1.07; *p* <.0001). Over three decades, the use of LLETZ as primary treatment for AIS increased (*p* <.0001, Table 1).

Up to 20 years follow‐up, 239 patients were diagnosed with high‐grade dysplasia (6.6%; 95% CI: 5.7–7.4) and 43 patients developed local cervical cancer (1.6%; 95% CI: 1–2.1) (Table 2). In total, 49 patients developed cervical cancer at any location (1.8%; 95% CI: 1.3–2.4), including 43 patients diagnosed with adenocarcinoma, five patients diagnosed with squamous cell carcinoma, and one patient diagnosed with cervical cancer not otherwise classified.

**TABLE 2:** 20‐year cumulative incidence of recurrent high‐grade cervical dysplasia, recurrent adenocarcinoma in situ (AIS), cervical intraepithelial neoplasia (CIN) grade 2–3, local cervical cancer, cervical cancer at any location, adenocarcinoma at any location, and squamous cell carcinoma at any location after primary treatment for AIS.

		Recurrent high‐grade cervical dysplasia				Recurrent adenocarcinoma in situ				Cervical intraepithelial neoplasia grade 2–3				Local cervical cancer			
		*n*	%	95%CI	Censor	*n*	%	95%CI	Censor	*n*	%	95%CI	Censor	*n*	%	95%CI	Censor
LLETZ	Radical	26	5.6	(3.3–7.9)	566	8	1.5	(0.4–2.5)	580	21	4.7	(2.5–6.8)	571	5	1.9	(0–4.4)	583
	Irradical	40	15.3	(10.4–19.9)	249	24	9.1	(5.2–12.9)	264	27	9.8	(6–13.4)	259	3	2.1	(0–4.6)	281
	Unclear	71	13.1	(10–16.2)	459	46	8.2	(5.8–10.5)	481	37	6.9	(4.5–9.2)	485	11	2.1	(0.8–3.3)	507
	Overall	137	10.5	(8.6–12.3)	1274	78	5.6	(4.3–6.9)	1325	85	6.6	(5.1–8.1)	1315	19	1.9	(0.8–3)	1371
CKC	Radical	64	4.7	(3.5–5.8)	1198	33	2.1	(1.4–2.8)	1221	36	2.9	(1.9–3.9)	1219	16	1.5	(0.7–2.3)	1231
	Irradical	20	9.9	(5.6–13.9)	140	15	7.5	(3.7–11.1)	142	8	4.0	(1.2–6.7)	148	6	3.1	(0.6–5.6)	147
	Unclear	18	7.6	(4.1–11)	141	13	5.6	(2.5–8.6)	144	8	3.2	(1–5.4)	148	2	1.5	(0–3.6)	151
	Overall	102	5.5	(4.4–6.6)	1479	61	3.1	(2.3–3.9)	1507	52	3.0	(2.2–3.9)	1515	24	1.7	(1–2.4)	1529
Hysterectomy		0	0.0	(0–0)	403	0	0.0	(0–0)	403	0	0.0	(0–0)	403	0	0.0	(0–0)	403
Overall		239	6.6	(5.7–7.4)	3156	139	3.6	(3–4.2)	3235	137	3.9	(3.2–4.6)	3233	43	1.6	(1–2.1)	3303

		Cervical cancer at any location^a^				Adenocarcinoma at any location			Squamous cell carcinoma at any location				
		*n*	%	95%CI	Censor	*n*	%	95%CI	Censor	*n*	%	95%CI	Censor
LLETZ	Radical	5	1.9	(0–4.4)	583	4	0.6	(0–1.2)	584	1	1.3	(0–3.8)	587
	Irradical	3	2.1	(0–4.6)	281	3	2.1	(0–4.6)	281	0	0.0	(0–0)	284
	Unclear	13	2.5	(1.1–4)	505	12	2.4	(1–3.8)	506	1	0.2	(0–0.5)	517
	Overall	21	2.1	(0.9–3.2)	1369	19	1.6	(0.8–2.3)	1371	2	0.5	(0–1.4)	1388
CKC	Radical	16	1.5	(0.7–2.3)	1231	13	1.2	(0.5–1.8)	1234	3	0.3	(0–0.8)	1244
	Irradical	7	4.2	(0.9–7.5)	146	7	4.2	(0.9–7.5)	146	0	0.0	(0–0)	153
	Unclear	3	2.3	(0–4.8)	150	2	1.5	(0–3.5)	151	0	0.0	(0–0)	153
	Overall	26	1.9	(1.1–2.7)	1527	22	1.5	(0.9–2.2)	1531	3	0.3	(0–0.6)	1550
Hysterectomy		2	0.6	(0–1.6)	401	2	0.6	(0–1.6)	401	0	0.0	(0–0)	403
Overall		49	1.8	(1.3–2.4)	3297	43	1.5	(1, 2)	3303	5	0.3	(0–0.6)	3341

*Note*: The colours give an idea of how high or low the cumulative percentage is.

Abbreviations: Censor, number of patients censored; CI, confidence interval; CKC, cold‐knife conization; high‐grade cervical dysplasia, adenocarcinoma in situ, or cervical intraepithelial neoplasia grade 2–3; LLETZ, large loop excision of the transformation zone; *n*, number of events; %, cumulative incidence of event.

^a^ One patient was diagnosed with cervical cancer not otherwise specified, 43 patients were diagnosed with adenocarcinoma, and five patients were diagnosed with squamous cell carcinoma.

**FIGURE 2:**
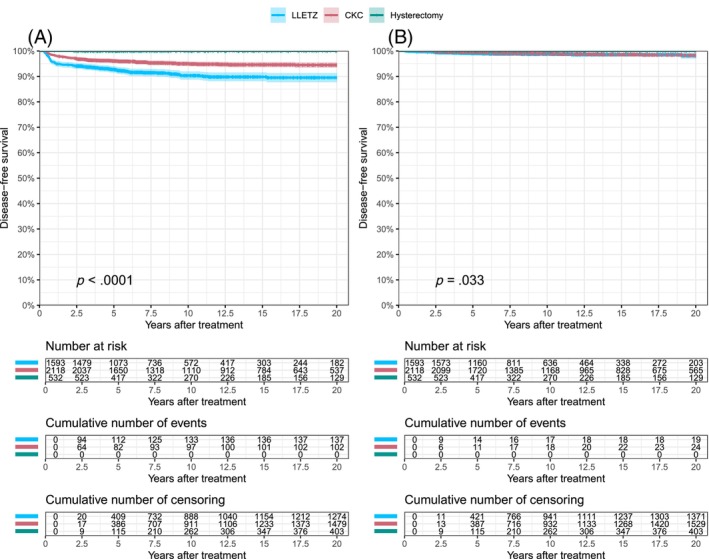
Disease‐free survival of recurrent high‐grade cervical dysplasia (A) and local cervical cancer (B) per treatment group.

Figure 2 and Table 2 show the disease‐free survival of high‐grade dysplasia and local cervical cancer after primary treatment. The incidence of high‐grade dysplasia and local cervical cancer after conservative treatment did not differ between the year intervals in which AIS was diagnosed (Supplemental Figure S3).

In the LLETZ group (*n* = 1593), 137 patients developed recurrent high‐grade dysplasia (cumulative incidence 10.5%; 95% CI: 8.6–12.3) and 19 patients developed local cervical cancer (1.9%; 95% CI: 0.8–3, Table 2). In the CKC group (*n* = 2118), 102 patients were diagnosed with recurrent high‐grade dysplasia (5.5%; 95% CI: 4.4–6.6) and 24 developed local cervical cancer (1.7%; 95% CI: 1–2.4, Table 2). After hysterectomy, none of the patients (*n* = 532) developed recurrent high‐grade dysplasia or local cervical cancer. However, two patients (0.6%) were diagnosed with distant cervical cancer after primary treatment by hysterectomy (Supplemental Table S2). Furthermore, one patient primarily treated by LLETZ, was diagnosed with distant cervical cancer after a hysterectomy during follow‐up (Supplemental Table S2).

The incidence of high‐grade dysplasia was higher after LLETZ compared to CKC (10.5% vs. 5.5%, *p* <.0001), while the incidence of local cervical cancer was similar between treatment groups (1.9% vs. 1.7%, *p* = .507). Compared to hysterectomy, the incidences of high‐grade dysplasia and local cervical cancer were higher after LLETZ (*p* <.0001 and *p* = .008, respectively) and after CKC (*p* <.0001 and *p* = .016, respectively).

**FIGURE 3:**
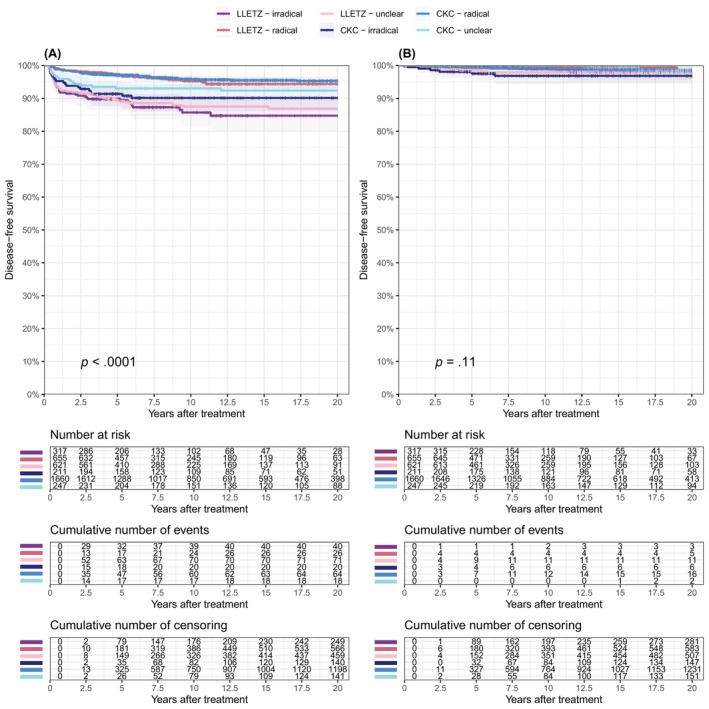
Disease‐free survival of recurrent high‐grade cervical dysplasia (A) and local cervical cancer (B) stratified by treatment (LLETZ and CKC) and surgical radicality.

For both conservative treatments, the risk of high‐grade dysplasia depended on surgical radicality (Figure 3). The incidence of high‐grade dysplasia after a radical LLETZ (5.6%; 95% CI: 3.3–7.9) was lower compared to an irradical LLETZ (15.3%; 95% CI: 10.4–19.9; *p* <.0001) and to an LLETZ with unclear radicality (13.1%; 95% CI: 10.0–16.2; *p* <.0001). The cumulative incidence of high‐grade dysplasia did not differ after an irradical LLETZ compared to an LLETZ with unclear radicality (*p* = .571). After CKC, the incidence of high‐grade dysplasia was lower after a radical excision (4.7%; 95% CI: 3.5–5.8) compared to an irradical excision (9.9%; 95% CI: 5.6–13.9; *p* = .0001) and compared to an excision with unclear radicality (7.6%; 95% CI: 4.1–11; *p* = .020). The cumulative incidence of high‐grade dysplasia did not differ after an irradical CKC compared to after a CKC with unclear radicality (*p* = .367).

The incidence of high‐grade dysplasia or local cervical cancer did not differ between LLETZ and CKC when a radical excision was achieved (*p* = .631 and *p* = .918, respectively), nor did it differ after irradical excision (*p* = .207 and *p* = .154, respectively). After a LLETZ with unclear radicality, the incidence of high‐grade dysplasia was higher than after CKC with unclear radicality (*p* = .041), but no difference was observed for the development of local cervical cancer (*p* = .186). Compared to hysterectomy, the incidence of high‐grade dysplasia was higher after a radical LLETZ (*p* <.0001) and after a radical CKC (*p* <.0001). Similarly, the development of local cervical cancer was higher after a radical LLETZ (1.9%; 95% CI: 0–4.4, *p* = .027) or radical CKC (1.5%; 95% CI: 0.7–2.3, *p* = .026) compared to hysterectomy (0%).

The relative hazard of high‐grade dysplasia was higher after LLETZ compared to CKC (HR 1.94; 95% CI: 1.50–2.51; *p* <.0001), also after adjustment in the first (HR 1.44; 95% CI: 1.10–1.88; *p* = .009) and second multivariable model (HR 1.45; 95% CI: 1.11–1.90; *p* = .007, Table 3). The mediation effect of radicality was 45.4%.

**TABLE 3:** Association between recurrent high‐grade dysplasia and development of local cervical cancer with treatment modality (LLETZ vs. CKC). Hazard ratios represent a comparison between LLETZ and CKC, with CKC as the reference category. The analyses included 3711 patients who received either a LLETZ (*n* = 1593) or a CKC (*n* = 2118).

	Recurrent dysplasia				Local cervical cancer			
	Coefficient	HR	95% CI	*p*‐value	Coefficient	HR	95% CI	*p*‐value
Crude	0.664	1.94	1.50–2.51	<.0001	0.205	1.23	0.68–2.23	.502
Model 1: adjusted for radicality	0.362	1.44	1.10–1.88	.009	0.002	1.00	0.54–1.87	.994
Model 2: adjusted for radicality, coexisting CIN lesions and age	0.370	1.45	1.11–1.90	.007	‐	‐	‐	‐

Abbreviations: CI, confidence interval; CIN, cervical intraepithelial neoplasia; CKC, cold‐knife conization; HR, hazard ratio; LLETZ, large loop excision of the transformation zone.

For the development of local cervical cancer, no difference was found after LLETZ compared to CKC in the univariable (HR 1.23; 95% CI: 0.68–2.23; *p* = .502) and multivariable model (HR 1.00; 95% CI: 0.54–1.87, *p* = .994, Table 3).

## 4 DISCUSSION

The 20‐year cumulative incidence of high‐grade dysplasia after treatment of AIS with LLETZ, CKC, and hysterectomy is 10.5%, 5.5%, and 0%, respectively (*p* <.0001). When a radical excision was achieved, recurrence rates of high‐grade dysplasia were similar between LLETZ and CKC (5.6% vs. 4.7%, *p* = .631). However, after primary treatment by LLETZ, the excision was less often radical compared to CKC (41.1% vs. 78.4%, *p* <.0001). Development of local cervical cancer did not differ after LLETZ or CKC (cumulative incidence 1.9% vs. 1.7%, *p* = .918), but was higher than after hysterectomy (cumulative incidence 0%, compared to LLETZ *p* = 0.008 and to CKC *p* = .016).

In current literature, there is controversy about the best conservative management of AIS, and studies comparing conservative treatment and hysterectomy are limited. Several studies have advocated CKC over LLETZ as safest conservative treatment for AIS,^7^, ^11^, ^14^ while others show comparable recurrence rates.^15^, ^16^ The most recent meta‐analysis of Jiang et al.,^13^ included 1559 patients of 18 retrospective studies and reported an AIS recurrence rate of 7.0% (*n* = 10/142) after LLETZ and 5.6% (*n* = 10/177) after CKC (*p* = .79). Furthermore, Baalbergen et al. reported that AIS recurrence rate after conservative treatment by CKC or LLETZ was not different from AIS recurrence after hysterectomy (*n* = 3/86 vs. *n* = 0/31, *p* = .56).^8^ This is in contrast with the findings of our study, which showed a higher incidence of high‐grade dysplasia, including recurrent AIS after LLETZ compared to CKC and after LLETZ and CKC compared to hysterectomy. This discordance could most likely be explained by our large study population and long follow‐up. Notably, previous studies only reported on AIS recurrences or development of adenocarcinoma, whereas we reported on recurrent CIN2‐3 and cervical cancer of other histotypes as well, because all (pre)malignant lesions are relevant for oncologic and obstetric outcomes.

We showed that the risk of high‐grade dysplasia was strongly associated with surgical radicality achieved after conservative treatment. This is in line with previous findings, which show recurrence rates of 17%–19.4% after positive surgical margins^7^, ^12^, ^28^, ^29^ and 2.6%–3% after negative margins.^7^, ^28^, ^30^ In our study, the incidence of high‐grade dysplasia was similar after a radical LLETZ compared to a radical CKC. This finding is consistent with previous studies, concluding that the safety of conservative treatment of AIS with LLETZ is comparable to CKC when a radical excision is achieved.^13^, ^31^ However, margins are believed to be more difficult to assess after LLETZ due to cautery artifacts, which could lead to unclear margins or false‐negative interpretations of margins.^1^, ^32^, ^33^ Our study reported a higher proportion of procedures with unclear radicality after LLETZ compared to after CKC. For both conservative procedures, higher recurrence rates were seen after excisions with unclear radicality compared to radical excisions, suggesting unclear margins are more likely to be in fact positive rather than negative for dysplasia.

The likelihood of irradical excision depends on the type of conservative procedure. In our cohort, excisions were irradical in 19.9% of patients (*n* = 317/1593) after LLETZ and in 10% of patients (*n* = 211/2118) after CKC. These rates are considerably lower than found in previous studies, showing positive margins of 44%–51% after LLETZ and 29%–30% after CKC.^7^, ^13^ However, both in our study and in these studies, the difference in rate of irradical excisions between LLETZ and CKC was significant. In a phase 2 pilot randomized controlled trial by Cohen et al., comparing LLETZ (*n* = 26) and CKC (*n* = 14) as treatment for AIS, no difference was seen in rate of incomplete excision for both procedures (15.7% vs. 28.6%, not significant).^34^ However, numbers were small, patients with lesions that were not amenable to a single‐pass excision were excluded and procedures were performed according to standardized protocol by clinicians knowing margin status would be compared. In our study, we analyzed margins only in the final specimen of primary treatment, as surgical radicality, which could explain the relatively low rates of incomplete excision compared to other studies reporting on margins status per surgical procedure. In our study population, two or more surgical excisions were performed in 10.0%, 28.8%, and 72.6% in the LLETZ, CKC, and hysterectomy groups, respectively.

Since the majority of patients with AIS are of reproductive age, reflected by a median age of 35 years at diagnosis in our study, obstetric outcomes after treatment are of utmost importance. Several studies showed an increased risk of preterm birth after cervical treatment.^17^, ^18^, ^19^ The risk of preterm birth was directly correlated with the dimension, that is, both depth and volume, of the excision and the number of treatments. When comparing conservative surgical procedures, the risk of preterm birth was higher after CKC than after LLETZ (10.8%–14.9% vs. 4.8%–8.1%).^18^, ^19^ In line with these findings, both modes of treatment and the omission of a second procedure, for example, in case of irradicality, should be considered in patients who still wish to conceive. The risk of recurrent high‐grade dysplasia may be acceptable in the light of increased risk of preterm birth after a subsequent treatment which further reduces cervical volume. However, intensive follow‐up with cervical cytology and high‐risk HPV testing is required to accept a higher risk for recurrence. A negative high‐risk HPV test and normal cytology seem reassuring with low risks of recurrent high‐grade dysplasia or cervical cancer.^35^, ^36^

To the best of our knowledge, this study is the largest study reporting on treatment strategy in patients diagnosed with AIS to date. Since this is a comprehensive, population‐based study, selection bias is assumed to be minimal and we could analyze all histologically proven recurrences. Of all included patients, a large and comprehensive number of pathology reports could be provided by Palga, resulting in a long median follow‐up.

Limitations of our study are the retrospective design and the lack of clinical data. Hence, the reason why patients with an irradical excision in our cohort were not re‐treated, is unknown. Furthermore, the lack of clinical data could lead to residual confounding. Another limitation was the variety in reporting details on treatment between clinicians over the years, which might have led to non‐differential misclassification of the primary treatment.

Future research directions might focus on the value of cytology and high‐risk HPV status during follow‐up in detecting recurrences and in optimizing individualized follow‐up schedules. Furthermore, a cost‐effectiveness analysis could provide more insights into the effectiveness and harms of performing a complementary hysterectomy after conservative treatment. Finally, future studies might investigate the risk of other high‐risk HPV‐associated (pre)malignant lesions of primary vulvar, anal, or vaginal origin after treatment for AIS.

## 5 CONCLUSION

The incidence of high‐grade dysplasia and local cervical cancer is similar after LLETZ or CKC when a radical excision for AIS is achieved. However, compared to CKC, LLETZ procedures are less often radical and have a higher chance of unclear radicality, which in turn is associated with higher recurrence rates. Omitting a complementary hysterectomy after completion of childbearing should be considered, given the favorable oncologic outcomes after radical excision. Independent of surgical radicality, no difference in development of cervical cancer was seen between both conservative treatments, but incidence of cervical cancer was lowest after hysterectomy. Both oncologic and obstetric outcomes should be weighted when counseling patients about their optimal treatment for AIS.

## AUTHOR CONTRIBUTIONS

**Mirte Schaafsma:** Investigation; writing – original draft; methodology; visualization; formal analysis; data curation. **Teska N. Schuurman:** Investigation; methodology; writing – original draft. **Pien Kootstra:** Data curation; writing – review and editing. **Deli Issa:** Writing – review and editing; data curation. **Ivo Hermans:** Writing – review and editing; data curation. **Maaike C. G. Bleeker:** Investigation; writing – review and editing. **Petra L. M. Zusterzeel:** Writing – review and editing. **Ruud L. M. Bekkers:** Investigation; writing – review and editing. **Albert G. Siebers:** Data curation; writing – review and editing. **Constantijne H. Mom:** Conceptualization; investigation; writing – review and editing; supervision; methodology. **Nienke E. van Trommel:** Investigation; conceptualization; writing – review and editing; supervision; methodology.

## CONFLICT OF INTEREST STATEMENT

The authors declare that they have no known competing financial interests or personal relationships that could have appeared to influence the work reported in this paper.

## ETHICS STATEMENT

According to the Dutch law, no ethical approval was required for this study.

## Supporting information


**Data S1.** Supporting Information.

## Data Availability

The data that support the findings of this study are available from the corresponding author upon request.
