# Supplementary material for: Nationwide cohort study on the risk of high‐grade cervical dysplasia and carcinoma after conservative treatment or hysterectomy for adenocarcinoma in situ
Source: Int J Cancer. 2024 Nov 4;156(6):1203–12. doi: 10.1002/ijc.35237 (PMC11736995; doi:10.1002/ijc.35237)
Supplement: Supplementary file 1 — Data S1. Supporting Information. [file IJC-156-1203-s001.docx]

**Supplementary appendix**

Nationwide cohort study on the risk of high-grade cervical dysplasia and carcinoma after conservative treatment or hysterectomy for adenocarcinoma in situ

Mirte Schaafsma^+^, Teska N. Schuurman^+^, Pien Kootstra, Deli Issa, Ivo Hermans, Maaike C.G. Bleeker, Petra L.M. Zusterzeel, Ruud L.M. Bekkers, Albert G. Siebers, Constantijne H. Mom^~^, Nienke E. van Trommel^~^

**Table of contents**

[1. Tables 2](#_Toc179464067)

[1.1. Supplemental table 1. 2](#_Toc179464068)

[1.2. Supplemental table 2. 2](#_Toc179464069)

[2. Figures 3](#_Toc179464070)

[2.1. Supplemental figure 1. 3](#_Toc179464071)

[2.2. Supplemental figure 2 4](#_Toc179464072)

[2.3. Supplemental figure 3. 5](#_Toc179464073)

# Tables

**1.1. Supplemental table 1.** General characteristics of patients stratified by year of AIS treatment.

|  |  | **1990-1999** | **2000-2009** | **2010-2019** | **2020-2021** | **p** |
| --- | --- | --- | --- | --- | --- | --- |
| Number of patients |  | 666 | 923 | 2,281 | 373 |  |
| Primary treatment (%) | LLETZ | 111 (16.7) | 317 (34.3) | 1,004 (44.0) | 161 (43.2) | <0.001 |
|  | CKC | 442 (66.4) | 495 (53.6) | 1,020 (44.7) | 161 (43.2) |  |
|  | Hysterectomy | 113 (17.0) | 111 (12.0) | 257 (11.3) | 51 (13.7) |  |
| Radicality (%) | Radical | 451 (67.7) | 626 (67.8) | 1,475 (64.7) | 272 (72.9) | 0.006 |
|  | Not radical | 71 (10.7) | 98 (10.6) | 323 (14.2) | 37 ( 9.9) |  |
|  | Unclear | 144 (21.6) | 199 (21.6) | 483 (21.2) | 64 (17.2) |  |

*Abbreviations: CKC: cold-knife conisation; LLETZ: large loop excision of the transformation zone; SD: standard deviation.*

**1.2. Supplemental table 2.** Description of patients who developed cervical cancer after primary treatment by hysterectomy for AIS.

| **Patient** | **Age** | **Primary treatment** | **Event** | **Location of recurrence** | **Time to recurrence in years** | **Description** |
| --- | --- | --- | --- | --- | --- | --- |
| #1 | 42 | Hysterectomy | Distant cervical cancer | Ovary | 13 | Adenocarcinoma in the ovary, positive for high-risk HPV type 16. Revision of the hysterectomy specimen supported AIS diagnosis and no invasive lesion was observed. |
| #2 | 60 | Hysterectomy | Distant cervical cancer | Ovary | 2.9 | Adenocarcinoma in the ovary, positive for high-risk HPV type 45. Revision of the hysterectomy specimen supported AIS diagnosis and no invasive lesion was observed. |
| #3 | 50 | LLETZ | Distant cervical cancer | Abdominal wall | 8.7 | Hysterectomy performed for recurrent AIS 6 months after primary treatment. Tumor origin unclear, but cervical origin preferred by pathologist (HPV not available). |

*Abbreviations: AIS: adenocarcinoma in situ; HPV: human papillomavirus; LLETZ: large loop excision of the transformation zone.*

# Figures


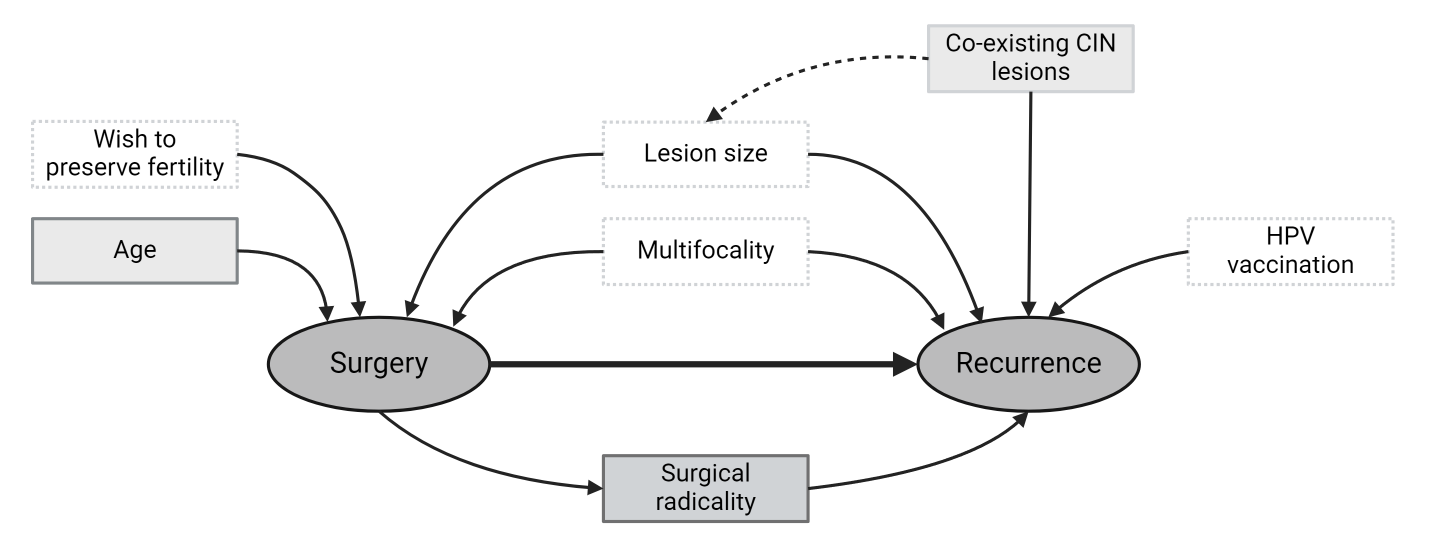


## **2.1. Supplemental figure 1.**

Directed acyclic graph of association between treatment and AIS recurrence. White boxes represent factors which are unknown in our study.


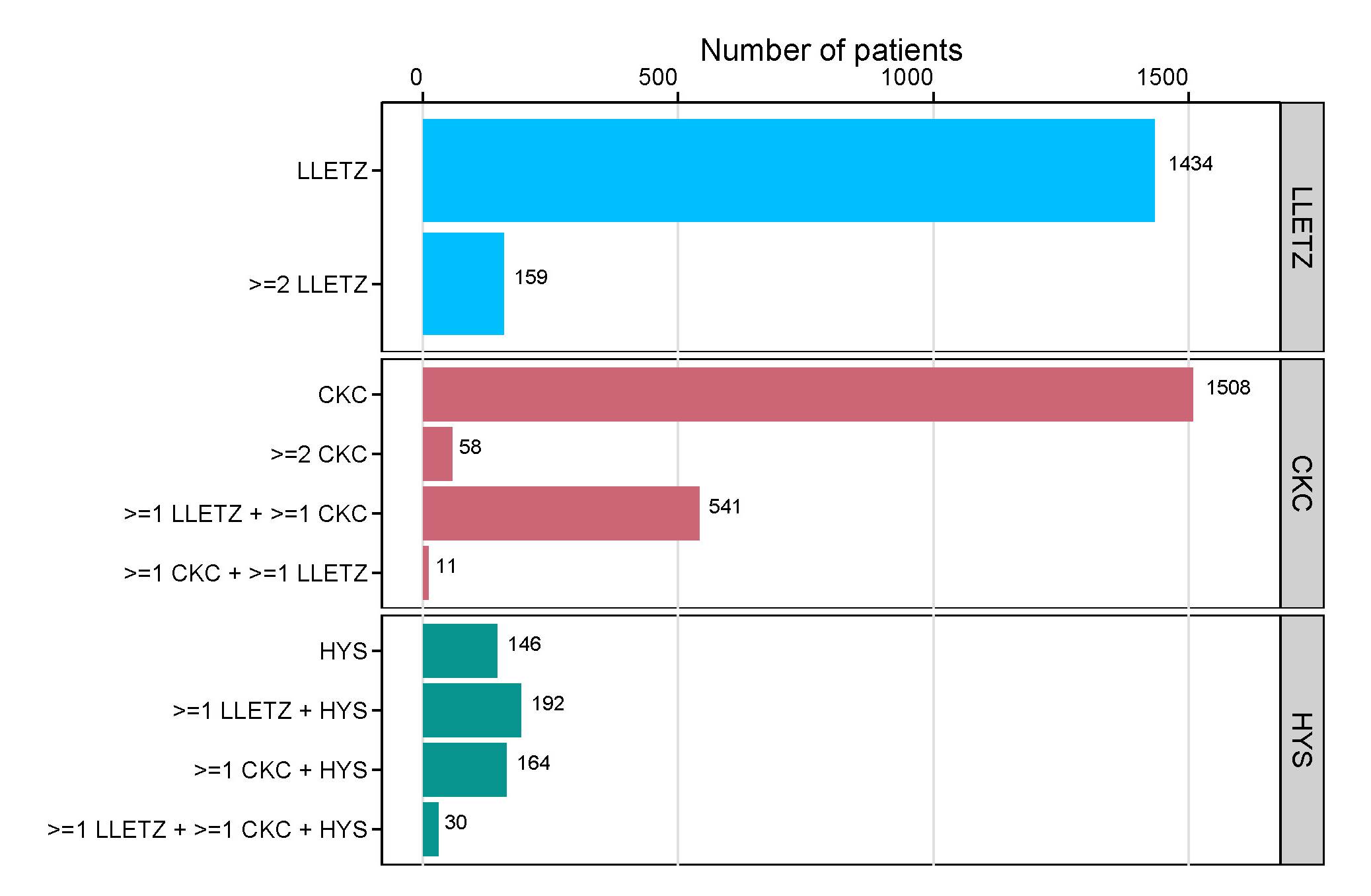


**2.2. Supplemental figure 2**. Number and type of surgical procedures per primary treatment group (categorized by most radical procedure). Abbreviations: LLETZ: large loop excision of the transformation zone, CKC: cold-knife conisation, HYS: hysterectomy.

*
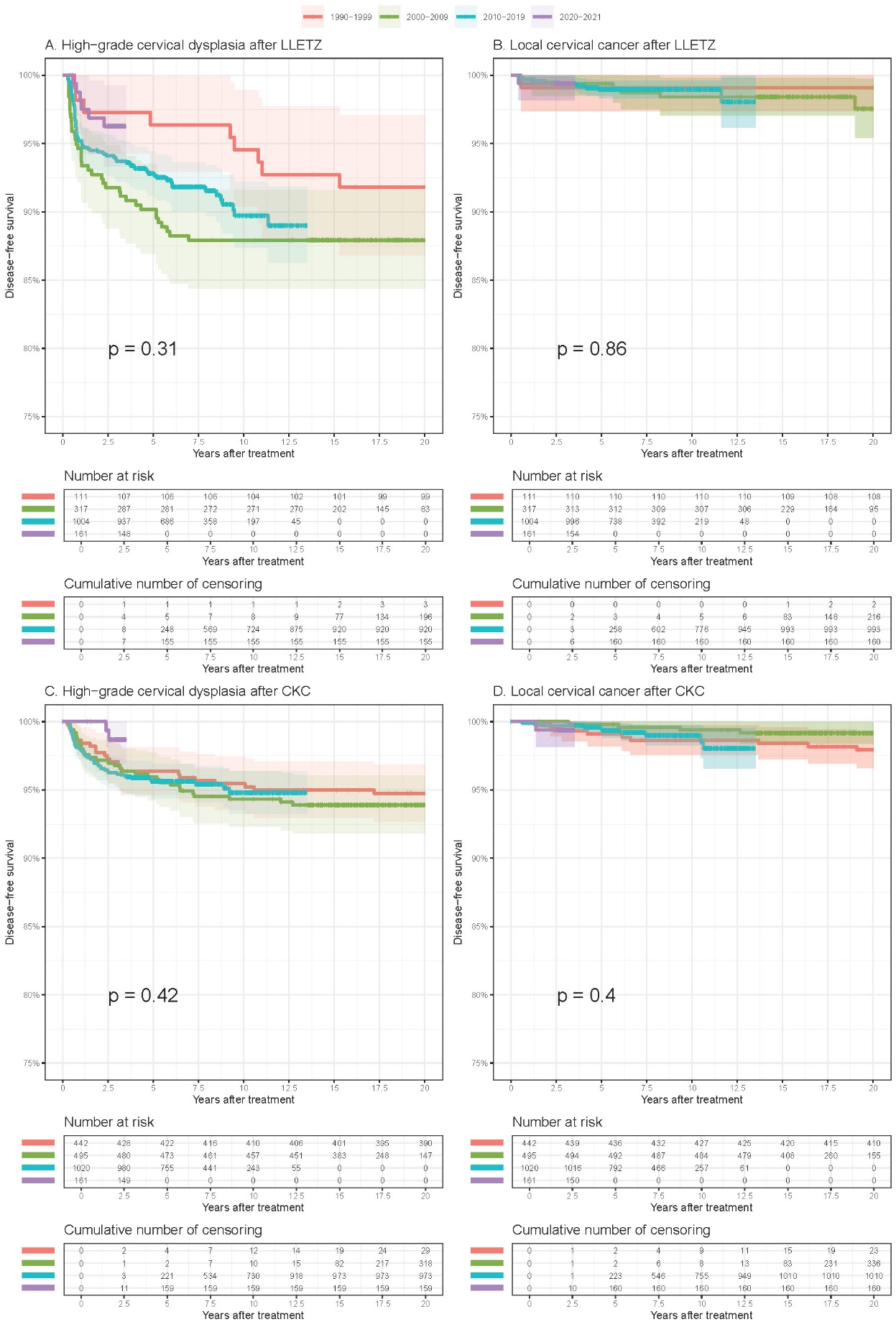
*

**2.3. Supplemental figure 3.** Disease-free survival of recurrent high-grade cervical dysplasia and local cervical cancer after LLETZ and CKC stratified by year of treatment. Note: the y-axis is zoomed in.
